# Comparison of tandem and single autologous stem cell transplantation in multiple myeloma: a retrospective propensity score-matching study

**DOI:** 10.1097/BS9.0000000000000235

**Published:** 2025-05-09

**Authors:** Shun-Quan Wu, Xiao-Fan Li, Zong-Jian Qiu, Zhi-Juan Zhu, Xian-Ling Chen, Ping Chen, Xiao-Hong Yuan, Rong Zhan, Nai-Nong Li

**Affiliations:** a Department of Hematology, Fujian Medical University Union Hospital, Hematopoietic Stem Cell Transplantation Center, Fujian Institute of Hematology, Fujian Provincial Key Laboratory on Hematology, Fuzhou, China; b Translational Medicine Center on Hematology, Fujian Medical University, Fuzhou, China

## To the Editor:

Upfront autologous stem cell transplantation (ASCT) has become the standard of care for transplant-eligible patients with newly diagnosed multiple myeloma (NDMM).^1,2^ Tandem ASCT, known as 2 planned sequential ASCTs within 3 to 6 months, was developed to improve overall survival (OS) in the conventional chemotherapy era, especially in patients who did not achieve a very good partial response (VGPR) after the first ASCT. However, in the era of novel agents, controversial data exist regarding the efficacy of tandem ASCT. Several studies have confirmed the superiority of tandem ASCT in prolonging progression-free survival (PFS) and OS, especially for patients with high-risk cytogenetics.^3–5^ In contrast, the results from the STaMINA (BMT CTN 0702) trial showed no PFS or OS benefits of tandem ASCT over single ASCT.^6^ Despite these conflicting findings, upfront tandem ASCT should be recommended for patients with MM and high-risk cytogenetics.^7,8^ Our previous study demonstrated that upfront tandem ASCT can overcome the unfavorable prognosis associated with International Staging System (ISS) stages and high-risk cytogenetics.^9^ However, data comparing single and tandem ASCT in patients with MM in China are still lacking. Thus, we conducted this retrospective propensity score-matching study to compare the clinical outcomes of tandem and single ASCTs in patients with NDMM treated at our center to help bridge the real-world knowledge.

All the ethical considerations strictly followed the Declaration of Helsinki. Informed consent was obtained from all patients. This analysis was approved by the institutional ethics committee (2021KY193).

We retrospectively reviewed our institutional database of transplant-eligible patients with NDMM who underwent ASCT between November 2014 and November 2021 and were followed up until April 2024. Eighty patients with NDMM who underwent ASCT were included in this study, with 40 patients in each group receiving either single or tandem ASCT. Matching was done for age, sex, subtype, β2-microglobulin, renal function, lactate dehydrogenase, ISS stages, cytogenetics, Revised-ISS, induction regimens, disease status at the time of transplant, year of transplant, and maintenance therapy regimen. The presence of t (4:14), t (14:20), t (14:16), amplification of 1q, or del (17p) by fluorescence in situ hybridization at the time of diagnosis was defined as high-risk cytogenetics. Response and disease progression were evaluated according to the International Myeloma Working Group consensus response criteria. Post-ASCT response was assessed at a maximum of 90 to 100 days after transplantation; thereafter, patients were followed up every 2 to 3 months. PFS duration was calculated from the date of the first transplant to disease progression or death. OS was defined as the time from the first transplant to death from any cause. Transplant-related mortality (TRM) was defined as death from any cause other than progression or relapse within 90 days and attributable to high-dose therapy. Absolute neutrophil count >500/μL and platelet >50,000/μL without transfusion support were defined as neutrophil and platelet engraftment, respectively.

SPSS software version 24 was used for propensity score matching and GraphPad Prism software version 9 was used for statistical analyses. The Kaplan–Meier method was utilized to calculate PFS and OS duration, and log-rank tests with 95% confidence intervals (95% CI) were used to compare time-dependent outcome measures. Statistical significance was set at *p* < 0.05.

The baseline characteristics of the patients receiving single or tandem ASCT are shown in Table 1. The 2 groups were evenly matched. A triplet induction regimen, including at least 1 novel agent, was administered to all patients for 4 to 6 cycles. Detailed treatment regimens are also available in Table 1. The EA (chemotherapy combination of etoposide and cytarabine; etoposide 100 mg/m^2^ qd d1–3 + cytarabine 0.5/m^2^ q12h d1–3) regimen,^10^ followed by granulocyte colony-stimulating factor, was used for stem cell mobilization and collection procedure. High-dose melphalan (140–200 mg/m^2^, depending on renal function) was administered as a conditioning regimen before ASCT. Single-agent lenalidomide was the most commonly administered maintenance treatment, followed by proteasome inhibitors (bortezomib or ixazomib).

**Table 1:** Characteristics of patients with NDMM receiving single and tandem ASCT.

Characteristics	Single cohort		Tandem cohort	
	Before PSM (n = 100)	After PSM (n = 40)	Before PSM (n = 50)	After PSM (n = 40)
Age (y), median (range)	54 (42–66)	55 (46–66)	53 (39–66)	53 (39–66)
Gender, n (%)				
Male	42 (42.0)	23 (57.7)	30 (60.0)	24 (60.0)
Female	58 (58.0)	17 (42.5)	20 (40.0)	16 (40.0)
Subtype, n (%)				
IgA	15 (15.0)	6 (15.0)	12 (24.0)	7 (17.5)
IgG	56 (56.0)	24 (60.0)	24 (48.0)	21 (52.5)
IgD	5 (5.0)	2 (5.0)	1 (2.5)	1 (2.5)
Light chain	24 (24.0)	8 (20.0)	13 (26.0)	11 (27.5)
ISS stage, n (%)				
I	34 (34.0)	13 (32.5)	20 (40.0)	15 (37.5)
II	36 (36.0)	14 (35.0)	15 (30.0)	12 (30.0)
III	30 (30.0)	13 (32.5)	15 (30.0)	13 (32.5)
R-ISS stage, n (%)				
I	32 (32.0)	10 (25.0)	14 (28.0)	10 (25.0)
II	38 (38.0)	22 (55.0)	25 (50.0)	22 (55.0)
III	30 (30.0)	8 (20.0)	11 (22.0)	8 (20.0)
Cytogenetics risk, n(%)				
Standard	55 (55.0)	16 (40.0)	20 (40.0)	16 (40.0)
High				
del (17p)	5 (5.0)	1 (2.5)	2 (4.0)	1 (2.5)
t (4;14)	4 (4.0)	1 (2.5)	3 (6.0)	1 (2.5)
t (14;16)	1 (1.0)	/	/	/
Amplification (1q)	23 (23.0)	12 (30.0)	15 (30.0)	13 (32.5)
Double/triple hit	12 (12.0)	10 (25.0)	10 (25.0)	9 (22.5)
Creatinine (μmol/L)				
Medium	103.6 (42–653)	104.5 (44–653)	101.5 (40–506)	102.6 (40–494)
>177, n (%)	14 (14.0)	4 (10.0)	5 (12.5)	5 (12.5)
Hemoglobin (g/L)				
Medium	92.5 (50–139)	97.7 (56–139)	94.5 (50–149)	96.8 (50–149)
<100, n (%)	52 (52.0)	20 (50.0)	22 (44.0)	19 (47.5)
Beta-2-MG (mg/L)				
Medium	4.6 (1.27–46.2)	5.4 (1.27–45.8)	4.7 (1.47–33.5)	5.2 (1.47–31.4)
>5.5, n (%)	29 (29.0)	11 (27.5)	15 (30.0)	10 (25.0)
LDH				
Abnormal, n (%)	22 (22.0)	7 (17.5)	14 (28.0)	8 (20.0)
Induction regimens, n (%)				
PAD-based	44 (44.0)	20 (50.0)	30 (60.0)	22 (55.0)
VRD-based	36 (36.0)	16 (40.0)	16 (32.0)	14 (35.0)
VTD/VCD-based	20 (20.0)	4 (10.0)	4 (8.0)	4 (10.0)
Status at transplant, n (%)				
sCR/CR	38 (38.0)	24 (60.0)	26 (52.0)	20 (50.0)
VGPR	52 (52.0)	12 (30.0)	17 (34.0)	14 (35.0)
PR	10 (10.0)	4 (10.0)	7 (14.0)	6 (15.0)
Time diagnosis-first transplant (mo), mean (range)	6.1 (4.1–10.9)	6.3 (4.3–10.9)	6.3 (4.3–11.9)	6.2 (4.3–11.7)
Time from first to second transplant (mo), mean (range)	/	/	4.0 (3.1–5.7)	4.0 (3.1–5.3)
Consolidation therapy, n (%)	3 (7.5)	3 (7.5)	4 (10.0)	4 (10.0)
Maintenance treatment				
Thalidomide	9 (9.0)	3 (7.5)	2 (4.0)	2 (5.0)
Lenalidomide	68 (68.0)	28 (70.0)	34 (68.0)	27 (67.5)
Bortezomib	10 (10.0)	4 (10.0)	4 (8.0)	4 (10.0)
Ixazomib	8 (8.0)	3 (7.5)	6 (12.0)	4 (10.0)
Lenalidomide + bortezomib	5 (5.0)	2 (5.0)	4 (8.0)	3 (7.5)

ACST = autologous stem cell transplantation, CR = complete response, LDH = lactate dehydrogenase, NDMM = newly diagnosed multiple myeloma, PAD = bortezomib, liposome doxorubicin, and dexamethasone, PR = partial response, PSM = propensity score-matching, sCR = stringent complete response, VGPR = very good partial response, VRD = bortezomib, lenalidomide, and dexamethasone, VTD/VCD = bortezomib, thalidomide/cyclophosphamide, and dexamethasone.

**Figure 1.**
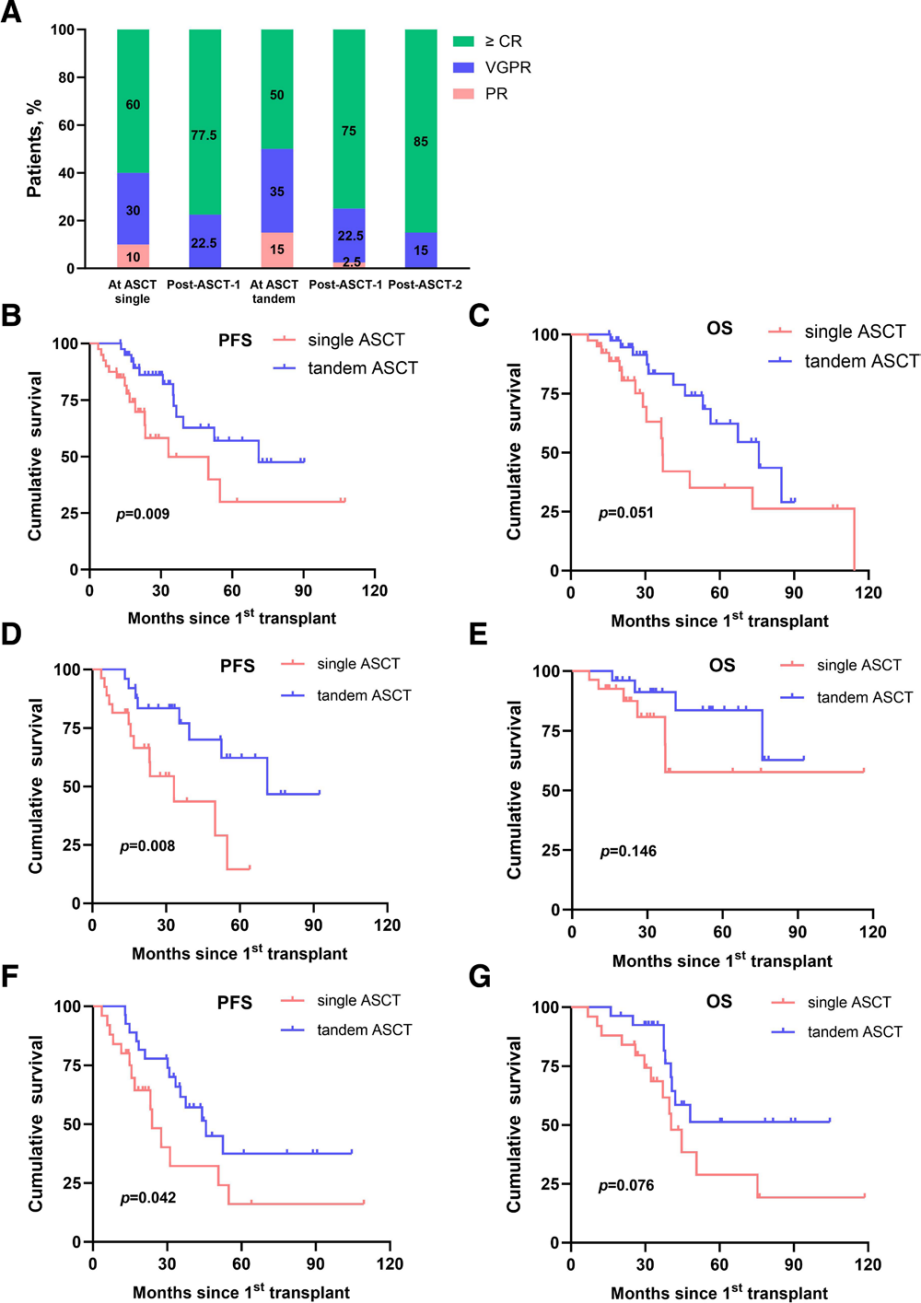
Response rate and cumulative incidences of PFS and OS in both groups of patients. (A) Response rate before and after ASCT. (B) PFS and (C) OS from transplant in patients undergoing single or tandem ASCT. (D) PFS and (E) OS from transplant in patients with ISS II/III receiving single (n = 27) or tandem (n = 25) ASCT. (F) PFS and (G) OS from transplant in patients with high-risk cytogenetics undergoing single (n = 24) or tandem (n = 24) ASCT. ASCT = autologous stem cell transplantation, CR = complete remission, ISS = International Staging System, OS = overall survival, PFS = progression-free survival, PR = partial response, VGPR = very good partial response.

The response rate after ASCT is shown in Figure 1A. After the last transplant, all patients achieved VGPR or better in both arms. In particular, 31 patients (77.5%) in the single ASCT group achieved greater than or equal to complete remission (CR) after ASCT, while 8 patients (20%) exhibited improved response after ASCT. In the tandem ASCT group, 30 patients (75%) achieved greater than or equal to CR after the first ASCT, while 9 patients (22.5%) exhibited improved response after the first ASCT. In addition, 10 patients (25%) in the tandem ASCT cohort further improved their response after the second transplant, while 34 patients (85%) achieved greater than or equal to CR after the second ASCT.

At data cutoff, with a median follow-up of 36.2 months (range: 12.9–116.4 months) from transplant, median PFS was 33.2 months (95% CI, 31.6–70.3) in the single ASCT group and 71.7 months (95% CI, 51.8–74.9) in the tandem ASCT group (*p* = 0.009, **Fig. 1B**). Median OS from the time of ASCT was 39.3 months (95% CI, 36.4–74.3) for the single ASCT cohort and 75.8 months (95% CI, 57.1–76.7) for the tandem ASCT cohort (*p* = 0.051, **Fig. 1C**). The estimated 7-year PFS and OS from the time of ASCT were 29.9% and 43.5%, respectively, for patients in the single ASCT group, compared with 47.5% and 54.4%, respectively, in the tandem group.

Furthermore, univariate analysis was performed to investigate the effect of single or tandem ASCT on the PFS and OS of patients with ISS Stage II/III at diagnosis and high-risk cytogenetics. Median PFS in patients with ISS II/III who underwent single ASCT was 29.8 months (95% CI, 16.3–32.9), compared with 66.4 months (95% CI, 32.1–71.4) in the tandem ASCT group of patients (*p* = 0.008) (**Fig. 1D**). Median OS from the time of transplant was not reached in either group (*p* = 0.146) (**Fig. 1E**). According to cytogenetics, median PFS in patients who exhibited high-risk cytogenetics was 23.2 months (95% CI, 10.3–44.1) in the single ASCT cohort and 43.4 months (95% CI, 23.9–55.3) in the tandem ASCT cohorts of patients (*p* = 0.042) (**Fig. 1F**). Patients with high-risk cytogenetics who received single ASCT had a median OS of 37.1 months (95% CI, 16.1–50.9), compared with not reached in the tandem ASCT group of patients (*p* = 0.076) (**Fig. 1G**). Multivariate analysis revealed no significant differences in PFS and OS based on disease stages and cytogenetics between the 2 groups.

A substantial number of studies have demonstrated that ISS stages and high-risk cytogenetics exert adverse prognostic impacts on PFS and OS. Tandem ASCT was used to improve the depth of response and long-term outcomes during the chemotherapy era. However, as mentioned above, different phase 3 trials exploring the role of tandem transplants have yielded mixed results, and the clinical value of tandem ASCT in patients with MM remains controversial in the novel agent era.^11^ Nevertheless, current recommendations support considering tandem ASCT for patients with MM and high-risk cytogenetics,^7,12^ especially in China, due to the lower availability of novel drugs. Previously, our single-center real-world study indicated no significant difference in PFS and OS based on ISS stages and cytogenetic risk in patients with MM undergoing tandem ASCT, suggesting that high-risk patients with myeloma could benefit from tandem transplants.^9^ However, the lack of a comparison with single ASCT is a major limitation of our previous study. Herein, this retrospective case–control study showed that patients with MM who underwent tandem ASCT had a greater than or equal to CR rate and longer PFS than those who underwent a single ASCT. Furthermore, tandem ASCT improved PFS in patients with advanced ISS stages and high-risk cytogenetics, supporting our previous conclusion concerning the role of tandem ASCT. This real-world study further strengthens the role of tandem ASCT for patients harboring high-risk disease characteristics and cytogenetics. These findings are consistent with those of a recent study by Dou et al,^13^ which indicates that tandem ASCT provides potential benefits for patients with advanced stages. However, patients with ISS III are often prone to renal impairment, limiting the benefits of tandem ASCT.^14^ Therefore, tandem ASCT should be considered with caution in such patients.

This study also found low toxicity associated with tandem ASCT in real-world cases, with a day 100 TRM of 0% in both cohorts. No significant differences were observed between the single and tandem cohorts regarding non-hematological toxicities. Oral mucositis, engraftment syndrome, and respiratory tract or intestinal infections were common but manageable complications. In the single arm and after the first transplant of tandem arm, median time to neutrophil engraftment was 11 days (range: 10–15) and 10.5 days (range: 9–14), respectively, while median time to platelet engraftment was 13 days (range: 10–17) and 13 days (range: 9–20), respectively. For patients who received a second ASCT in the tandem arm, the median times to neutrophil and platelet engraftment were 12 days (range: 10–25) and 17 days (range: 9–62), respectively. The median duration of antibiotic therapy was 7 days in the single arm and the first transplant in the tandem arm (range: 5–10 and 6–11, respectively). The median duration of antibiotic therapy was 8 days (range: 6–12) in the second transplant of the tandem arm. One patient without prior exposure to lenalidomide developed a second primary malignancy, namely, acute lymphoblastic leukemia, 4.5 years after the second transplant.

Several considerable limitations of the current study include its retrospective nature and relatively small sample size. In addition, our analysis included patients who were previously treated between 2014 and 2021. Therefore, the significant variation in the follow-up time represents another limitation, and clinical outcomes might have changed over the follow-up period.

In conclusion, tandem ASCT was associated with a greater than or equal to CR rate and longer PFS than single ASCT, with a trend toward longer OS, particularly among patients with MM who have advanced ISS stages and high-risk cytogenetics. Moreover, similar to single ASCT, tandem ASCT may be tolerated by patients with MM. However, the real-world evidence presented here has several limitations. At present, an increasing number of novel drugs, including anti-CD38 monoclonal antibodies, bispecific antibodies, immunomodulatory agents, and newer-generation proteasome inhibitors, have become available for treating patients with NDMM. Therefore, further studies comparing the efficacy and tolerability of tandem ASCT with single ASCT are essential.

## ACKNOWLEDGMENTS

This work was supported by the joint funds for the innovation of science and technology, Fujian province (2021Y9050), Fujian provincial health technology project (2021ZD01005). This work was also sponsored by Fujian province science and technology major special project (2022YZ034016).

## AUTHOR CONTRIBUTIONS

N.-N.L. and R.Z. conceptualized the study design. S.-Q.W., X.-F.L., Z.-J.Q., Z.-J.Z., X.-L.C., P.C., and X.-H.Y. conducted data collection and managed the patients. S.-Q.W. and X.-F.L. were responsible for data curation, analyzed the data, performed statistical analyses, and drafted the manuscript. N.-N.L. reviewed the manuscript. All authors contributed to the article and approved the final manuscript.
